# Inflammatory and Redox Blood Gene Expression Fingerprint of Obstructive Sleep Apnoea in Patients with Alzheimer’s Disease

**DOI:** 10.1002/alz.087240

**Published:** 2025-01-09

**Authors:** Leila Romero ElKhayat, Faride Dakterzada, Raquel P Huerto, Anna Carnes, Adriano Targa, Ferran Barbe, Elena Milanesi, Antonio Cuadrado, Gerard Piñol‐Ripoll

**Affiliations:** ^1^ IRB LLeida, LLeida Spain; ^2^ Hospital Universitari Santa Maria de Lleida, IRBLleida, Lleida Spain; ^3^ Hospital Universitari Santa Maria, LLeida Spain; ^4^ Cognitive Disorder Unit, Hospital Universitari Santa Maria, Lleida Spain; ^5^ IRBLleida, Group of Translational Research in Respiratory Medicine, Lleida, Spain, Lleida Spain; ^6^ Hospital Universitari Santa Maria LLeida, LLeida Spain; ^7^ “Victor Babes” National Institute of Pathology, Bucharest Romania; ^8^ Department of Biochemistry, Faculty of Medicine, Autonomous University of Madrid, Madrid Spain

## Abstract

**Background:**

Obstructive sleep apnoea (OSA) is the sleep disorder most frequently found in patients with Alzheimer’s disease (AD). The intermittent hypoxia (IH) caused by OSA may participate in AD pathogenesis through increase in oxidative damage and inflammation. We aimed to identify inflammatory and redox genes differentially expressed in the blood from AD patients with severe OSA compared with those with nonsevere OSA.

**Method:**

We included 40 AD patients diagnosed based on clinical manifestations and AD biomarker levels in cerebrospinal fluid (CSF). Severe or nonsevere OSA (apnoea–hypoapnoea index ³ 30/h and < 30/h, respectively) was diagnosed through overnight polysomnography (PSG). The expression levels of 136 inflammation‐related and 84 redox‐related genes were evaluated by whole blood targeted transcriptomics.

**Result:**

We identified three inflammatory genes and six redox genes that were upregulated in AD patients with severe OSA. Of all upregulated genes, TNFSF14 and ALOX12 (inflammatory and redox genes, respectively) were positively correlated with the arousal index. GSR, a redox gene, was positively correlated with the AHI (p<0.05). Finally, we found good correlations between the three inflammatory genes and six redox genes (p<0.05). A pathway enrichment analysis showed a strong enrichment of the serotonergic synapse pathway in severe OSA AD patients.

**Conclusion:**

Our results show an upregulation of nine genes involved in NF‐κB‐mediated inflammation and redox metabolism in the blood of patients with mild AD with severe OSA. Therefore, OSA may worsen the inflammation and oxidative damage that are already altered in patients with AD.

